# Clinical profile and outcome of acute organophosphate poisoning in children of Upper Egypt: a cross-sectional study

**DOI:** 10.1186/s12887-021-02563-w

**Published:** 2021-02-26

**Authors:** Khaled A. Abdel Baseer, Eman Fathala Gad, Yaser F. Abdel Raheem

**Affiliations:** 1 Department of Pediatrics, Qena Faculty of Medicine, South Valley University, Qena, Egypt; 2 Department of Pediatrics, Faculty of Medicine, Assiut University, Assiut, Egypt

**Keywords:** Organophosphates poisoning, Mortality, Pattern, Outcome

## Abstract

**Background:**

Organophosphates are one of the most common agents of poisoning in developing countries including Egypt. Due to lack of data about characteristics of organophosphates poisoning in our localities, we aimed to evaluate its clinical pattern and factors affecting outcome.

**Methods:**

It was a cross-sectional study conducted in South valley University hospital between January 2019 and December 2019. It included all children ≤16 years of age presented with organophosphates poisoning. Diagnosis was performed from the history taken from the patient’s relatives and presenting symptoms. Demographic data, mode and route of poisoning, time from exposure to presentation, clinical symptomatology, grading and routine investigations were evaluated in addition to treatment taken and outcome.

**Results:**

During the study period, 108 children; mean age was 7.95 ± 4.11 years (range 1. 5-16 years) presented with organophosphorous poisoning. Sixty five (60%) cases were females and 43 (40%) were males. Unintentional acts (87%) were detected more than suicidal (13%) and inhalation route (63.8%) more than ingestion (36. 2%). Miosis was the most frequent clinical sign (100%) followed by respiratory distress (77.7%). Regarding time of presentation to emergency department, 43 (40%) cases were presented within 6 h while others presented more than 6 h post-exposure. Mechanical ventilation (MV) was needed for 14 (13%) cases and 6 (5.5%) cases died due to respiratory failure. Duration of hospital stay, mean time interval from toxic exposure to hospital presentation, leucocytosis, need for MV and cumulative dose of pralidoxime were significantly higher in non survivors than survivors while Pao2 (partial arterial oxygen) and GCS (Glasgow coma scale) were significantly lower.

**Conclusion:**

This study concluded that time consumed till presentation to hospital, low GCS, need for MV, leucocytosis, decreased PaO2 and increased cumulative dose of pralidoxime were independent risk factors of mortality.

**Supplementary Information:**

The online version contains supplementary material available at 10.1186/s12887-021-02563-w.

## Background

Organophosphate compounds (OPCs) are acetylcholinesterase (AChE) inhibitors used as pesticides with a potential for induction of systemic illness upon toxic exposure [1, 2]. Due to their easy availability and low cost, organophosphates are one of the most common causes of poisoning in the world from agricultural, unintentional, or suicidal exposure [3]. These compounds enter the human body by ingestion, inhalation or skin absorption, and irreversibly inhibit AChE that is responsible for degradation of acetylcholine (Ach) [4, 5]. Accumulation of Ach activates muscarinic and nicotinic receptors at synapses within the peripheral and central nervous systems producing neurotoxic sequelae with a high mortality rate [6]. The initial management of acute OP poisoning includes cardio-respiratory stabilization and decontamination including removing clothes (a possible source of continued exposure), washing of skin and eyes, and consideration of gastric lavage and activated charcoal [7]. Atropine which is peripheral and central muscarinic receptor antagonist as well as pralidoxime chloride, which reactivates inhibited AChE are the mainstay of treatment [8]. Most deaths occur due to respiratory and cardiovascular failure, respiratory muscles paralysis and obstruction caused by bronchial secretions and OP induced bronchospasm [9]. Due to paucity of research on the clinical characteristics and outcome of poisoning in children of our region, this study was conducted with the aim of evaluating clinical pattern and risk factors of mortality by organophosphates poisoning.

## Methods

It was a cross-sectional study conducted in South valley University hospital, Egypt between January 2019 and December 2019. It included all children <16 years of age presenting to emergency and intensive care units with organophosphate poisoning. Data was collected from parents or care-givers using a pre–determined questionnaire (Additional file 1). The baseline demographics including age, gender and residence in addition to clinical data were recorded for all recruited patients. Detailed history and basic information related to circumstances of poisoning were taken including mode of poisoning, time of exposure, reason of poisoning, type of poison and poisoning route. The symptoms with which the patients presented were recorded and detailed clinical examination including Glasgow coma scale (GCS) was done. On admission to the Emergency Department, clinical classification and grading of organophosphorous poisoning as estimated by Bardin et al. [10], was used for evaluation of intoxication. They classify cases into grade 1 as having hypersecretion, fasciculations and conscious, grade 2 as Grade 1 with hypotension but unconscious and Grade 3 as Grade 2 with stupor, abnormal chest x-ray and paO2 < 10 mmHg. This grading is of great value in recognition of severely intoxicated children who need rapid admission to the intensive care unit. Generally, as our rural locality depends mainly on agriculture, organophosphate compounds are in common use and frequently found in the home.

Poisoned cases are usually diagnosed on the basis of history of exposure or contact and characteristic clinical picture specially presence of pin point pupil, excessive chest secretions and distinct garlic odor of the patient’s breath and vomits that characterize most of these compounds. In doubtful cases, a therapeutic trial of atropine is employed, with a good response to atropine supporting the diagnosis. However, In our population, the bottle of consumed OP compound often accompanies the patient to the Emergency Department. If a patient did not present with a clinical history or clinical features consistent with OP poisoning, they were excluded from the study.

Baseline investigations included complete blood count, urea, creatinine and arterial blood gas values. After diagnosis, patients underwent immediate assessment and care of airway, breathing, and circulation and those admitted with ingestion were subjected to gastric lavage followed by repeated doses of activated charcoal. In those of inhalation and dermal exposure, all the clothes were replaced and the skin washed with water and soap. All patients were given atropine initially 0.02 mg/kg intravenously. The main end points of atropinization were a clear chest on auscultation with resolution of crepitations and wheeze and a heart rate more than 80 beats/min. If these targets were not achieved by 3-5 minutes, we doubled the intravenous dose continuously every 3-5 minutes until atropinization had been achieved.

Due to its high cost, pralidoxime is generally reserved for patients with evidence of severe nicotinic receptor stimulation such as muscle fasciculations and progressive muscle weakness. It was administered as loading dose of 30mg/kg IV over 30 minutes (maximum 1 g), followed by an infusion of 10 mg/kg/h (maximum rate 650 mg/hr) for 2-4 days. Atropine was used at same time with pralidoxime. The institutional ethics committee of South valley University approved the study and parents provided informed consent.

### Statistical analyses

Statistical analyses of the data collected from the two outcome groups (survivors and non survivors) were compared using SPSS version 20 for Windows (Chicago, IL. USA). Continuous variables were expressed as mean ± standard deviation, and categorical variables were expressed as frequency. Fisher exact test was used examine the relation between qualitative variables and t-test used for comparison of means. Linear regression analysis was used to study significant predictors of outcome. A p value < 0.05 was considered statistically significant.

## Results

Sociodemographic and laboratory characteristics of studied patients were summarized in Table 1. The mean age of patients was 7.95±4.11 years (range 1. 5-16 years). Sixty five (60%) children were females with female to male ratio of 1.5: 1. Unintentional acts were more than suicidal and poisoning by inhalation route more than by ingestion. Exposure patterns showed that spraying for pest control in the field was the most frequent source followed by lotions used for treatment of head lice that are usually home made using OPC. Mean time (hours) from poisoning exposure to emergency unit presentation was 6.06± 3.12 (range 1-36 hours) and mean duration (days) of hospital stay was 3.51± 1.75 days (range 2-10 days). Of the known documented (96) cases, most poisonings were by malathion (75%), followed by Chlorpyrifos (8. 3 %) and diazinon (4. 2%). Mortality rate was 5.5% due to respiratory failure. Table 2 compares poisoned children according to mode of intoxication. Suicidal attacks were significantly more common in older children and females. Duration of hospital stay was significantly more prolonged with suicidal attempts. Clinical and therapeutic features of patients were shown in Table 3. Forty three (40%) cases were severely poisoned and 29 (26.8%) were in deep coma. Miosis was the most common clinical feature(100%) followed by respiratory distress (77.7%), vomiting (43.5%) and bradycardia (33. 3%). The most commonlyencounteredcomplications were respiratory failure (13%) followed by aspiration pneumonia (4.6%). Mechanical ventilation was needed in 14 (13%) cases due to respiratory failure. All patients were given atropine initially at emergency department (0. 2-0.8mg) with cumulative dose 1.33±0.52 mg. Pralidoxime chloride was used in 9 cases only initially at emergency department (0. 5-1gm) with cumulative dose 13.95±5.04 gm. Table 4 compares poisoned patients according to outcome. Duration of hospital stay, mean time interval from toxic exposure to hospital presentation, leucocytosis and need for mechanical ventilation were significantly higher in non survivors than survivors. On the other hand, Pao2 and GCS were significantly lower in non survivors. Mortality was more with Chlorpyrifos followed by diazinon and least with malathion. Multivariate regression analyses (Table 5) revealed that time consumed till presentation to hospital, low GCS, need for MV, leucocytosis, decreased PaO2 and increased cumulative dose of pralidoxime were independent risk factors of mortality.

**Table 1:** Demographic and laboratory characteristics of studied patients

Variable	Total patients = 108 Mean ± SD/n (%)
Age (years)	7.95 ± 4.11
Sex	
Male	43 (40%)
Female	65 (60%)
Residence	
Urban	35 (32.5%)
Rural	73 (67.5%)
Mode of poisoning	
Unintentional	94 (87%)
Suicidal	14 (13%)
Route of poisoning	
Inhalation	69 (63.8%)
Ingestion	39 (36. 2%)
Identifiable sources of poisoning	
Exposure in agriculture field	41 (38%)
Lotion for head lice	33 (30.5%)
Surface & room sprays	20 (18.5%)
Spray for house insects	14 (13%)
Type of poisoned organophosphate	
Malathion	81 (75%)
Chlorpyrifos	9 (8. 3%)
Diazinon	6 (5.5%)
Unknown	12 (11. 2%)
Outcome	
Improved	102 (94.5%)
Expired	6 (5.5%)
Blood picture	
Hemoglobin, g/dL	10.14 ± 1.52
Leucocytes, × 10^3^/mL	10.56 ± 3.24
Platelets × 10^3^/mL	310.11 ± 89.20
Blood gases	
PaO2 mmHg	96.46 ± 11.48
PaCO2 mmHg	40.21 ± 3.87
Duration of hospital stay (days)	3.51 ± 1.75
Time to presentation (hours)	6.06 ± 3.12
Glasgow coma scale	(Median= 13, IQR = 7)

*PaO2* Partial pressures of oxygen, *PaCO2* Partial pressures of carbon dioxide, *IQR* Interquartile range

**Table 2:** Comparison between studied cases according to mode of poisoning

Variables	Unintentional *N* = 94	Suicidal *N* = 14	*P* value
Age (years)			
≤ 6 years (*n* = 46)	46 (48.9%)	0 (0%)	< 0.001
> 6 - < 12 years(*n* = 27)	27 (28.7%)	0 (0%)	
≥ 12 years(*n* = 35)	21 (22.4%)	14 (100%)	
Sex			
Males (*n* = 43)	41(43.6%)	2 (14. 3%)	< 0.05
Females (*n* = 65)	53 (56.4%)	12 (85.7%)	
Residence			
Urban (*n* = 35)	29 (30.9%)	6 (42.9%)	NS
Rural (*n* = 73)	65 (69. 1%)	8 (57. 1%)	
Need for mechanical ventilation	10 (10.6%)	4 (28.6%)	NS
Duration of hospital stay (days)	3.24 ± 1.59	5.35 ± 1.73	< 0.01
Mortality	4 (4. 3%)	2 (14. 3%)	NS

*NS* Non significant; *P* value < 0.05 is significant

**Table 3:** Clinical and therapeutic features of studied cases

Variable	Total patients (*n* = 108) n (%)
Severity of poisoning	
Mild (grade 1)	19 (17.5%)
Moderate (grade 2)	46 (42.5%)
Severe (grade 3)	43 (40%)
Time to presentation	
≤ 6 h	43 (40%)
> 6 h	65 (60%)
Consciousness and brain injury status	
Conscious	42 (39%)
Mild brain injury (GCS 13–15)	24 (22. 2%)
Moderate brain injury (GCS 9-12)	13 (12%)
Sever brain injury (GCS ≤ 8)	29 (26.8%)
Common clinical manifestations at presentation	
Miosis	108 (100%)
Respiratory distress	84 (77.7%)
Vomiting	47 (43.5%)
Bradycardia	36 (33. 3%)
Sweating	17 (15.7%)
Convulsion	15 (13.8%)
Muscle weakness	12 (11. 1%)
Fasciculations	9 (8%)
Complications	
Respiratory failure	14 (13%)
Aspiration pneumonia	5 (4.6%)
Septicemia	3 (2.8%)
Acute renal failure	1 (0.9%)
Use of atropine (all cases)	
Initial at emergency	0. 2–0.8 mg
Cumulative dose	1.33 ± 0.52 mg
Use of pralidoxime (9 cases)	
Initial at emergency	0. 5-1g
Cumulative dose	13.95 ± 5.04 g
Need for Mechanical ventilation	14 (13%)
Decontamination (charcoal &gastric lavage)	39 (36. 1%)

*GCS* Glasgow coma scale

**Table 4:** Clinical and laboratorial variables between survivors and non survivors

Variables	Survivors (*n* = 102)	Non survivors (*n* = 6)	*P* value
Age (years)	8.09 ± 4.20	4.51 ± 1.04	< 0.05
Duration of hospital stay (days)	3.43 ± 1.72	5.16 ± 1.78	< 0.05
Time to presentation (hours)	5.91 ± 3. 05	8.66 ± 3.32	< 0.05
Glasgow coma scale	9.74 ± 1.26	7.50 ± 0.83	< 0.001
Need for mechanical ventilation	9/102 (8.8%)	5/6 (83. 3%)	< 0.001
Start treatment before arrival	26 (25.5%)	0/6 (0%)	< 0.001
***Type of poisoned organophosphate***			
Malathion (81 cases)	78 (96. 3%)	2 (3.7%)	< 0.001
Chlorpyrifos (9 cases)	7 (77.8%)	2 (22. 2%)	
Diazinon (6 cases)	5 (83. 3%)	1 (17.7%)	
Unknown (12 cases)	11 (91.6%)	1 (8.4%)	
Hemoglobin, g/dL	9.99 ± 1.59	9.56 ± 1.67	NS
Leucocytes, × 10^3^/mL	10.32 ± 3.09	14.66± 3.14	< 0.01
Platelets × 10^3^/mL	308.35 ± 89.98	340.88 ± 74. 61	NS
PaO2 mmHg	97.23 ± 11.31	83.33 ± 4.41	< 0.01
PaCO2 mmHg	40.18± 3.86	40.66 ± 4.27	NS
Cumulative dose of atropine (mg)	1.31 ± 0.52	1.61 ± 0.44	NS
Cumulative dose of pralidoxime (gm)	9.65 ± 3.68	17.4 ± 2.70	< 0.01

*PaO2* Partial pressures of oxygen, *PaCO2* Partial pressures of carbon dioxide; *P* value < 0.05 is significant; *NS* Non significant

**Table 5:** Multiple linear regression analysis of independent risk factors of mortality

Variable	β	Standard error	Hazard ratio	Confidence Interval 95%	*P* Value
Time to presentation (hours)	−0.127	0.030	0.881	0.831–0.934	< 0.001
Glasgow coma scale	0.036	0.006	1.037	1.025–1.048	< 0.001
Need for MV	0.158	.041	1.314	1.219–1.436	< 0.001
Duration of hospital stay (days)	0.008	0.019	1.008	0.971–1.047	NS
Leucocytes, × 10^3^/mL	0.169	.057	1.534	1.461–1.873	< 0.001
PaO2 mmHg	−0.033	0.008	1.033	1.017–1.049	< 0.001
Cumulative atropine dose (mg)	0.003	0.002	1.003	0.998–1.007	NS
Cumulative pralidoxime dose (gm)	0.187	0.039	1.205	1.115–1.302	< 0.001

*MV* Mechanical ventilation, *PaO2* Partial pressures of oxygen, *P* value significant < 0.05; *NS* Non significant

## Discussion

Organophosphates poisoning (OP) is a major health problem all over the world, particularly in the developing countries due to widespread usage and application of pesticides in agricultural and environmental pest control [11]. Easy availability and excessive popularity of its use as insecticides and pesticides has increased the incidence of ingestion, resulting in increasing suicidal and unintentional poisoning [12]. In the present study, the majority of organophosphates poisoning occurred due to unintentional ingestion or inhalation specially in young children. Therefore, it can be reduced through use of effective preventive measures and community health education.

Females showed an evident preponderance over males with male: female ratio of 1: 1.5. This finding was in agreement with studies conducted in Turkey (ratio of 1: 1.47) [13] and Nepal (ratio of 1: 2) [14] while in the study conducted by Banday et al. [15], male : female ratio was 1: 3. 2. This may be due to increased incidence of suicide in females being more sensitive and can be affected easily by emotional conflicts specially those more than 14 years.

Approximately two thirds of poisoned cases were from rural areas (67.5%) and this was consistent with other studies [16, 17]. As our country is dependent mainly on agriculture, pesticides are readily available at home and easily accessible by children, thereby exposing them to unintentional poisonings. Additionally, older children may work with parents in the farms, and are therefore more susceptible to dermal contact and inhalation of organophosphates used for pest control.

In agreement with Banday et al [15], miosis was the most frequently encountered sign in our study(100%) followed by respiratory symptoms while in Banerjee et al., study [18] miosis (91.94%) was the most common sign and vomiting was the most common symptom (85.02%). Variation of clinical presentation from one to another study may be related to the type OP involved, the quantity absorbed, and route of exposure.

The mortality following OP poisoning varies from 4-30% [19]. Mortality rate in our study was 5.5% which was comparable to a previous study done in Nepal (5.9%) [17]. Causes of death in our cases were related to respiratory failure in 4 cases and aspiration pneumonitis complicated bysepticemia in two. Delayed presentation to hospital owing to difficult transport from rural areas particularly for cases poisoned at night, delayed endotracheal intubation and improper preliminary resuscitation measures aggravate the problem. Some cases brought to our centers after ingestion of large amount of water and suffered repeated aspiration and severe electrolyte disturbance. Our finding of frequent deaths in younger than older children may be related to their small body mass giving higher body surface area in relation to small weight and therefore, more exposure. In general, pediatric studies report frequency of unintentional ingestions and tend to have a lower mortality than adult studies. As reported by Reddy et al. [20], and Ali et al [21] studies, adults are exposed to higher doses with intentional ingestions so carry higher mortality. The mean time to presentation from toxic exposure to hospital presentation in our study was significantly higher in non survivors. In concordance with our notice, the study done by El- Naggar et al. [22], estimated average time for admission to the emergency department ranging from one to 6 hours, and they concluded that rapid hospital admission was a vital factor for the low mortality rate of their studied cases. In the current study, 61% presented with altered sensorium; of them 26.8% were in deep coma with GCS ≤ 8.Levy-Khademiet al [23], reported neurological manifestations as the major clinical manifestation following organophosphate exposure in children and noticed coma and/or seizures in (71%) of studied cases. It was documented that OPC induce neuropathy leading to hypoperfusion in the central nervous system and this with other hemodynamic abnormalities causes the GCS value to decrease. Low values of GCS have the potential for development of respiratory failure and worse prognosis [24]. Mortality was also high in patients who required mechanical ventilation than others. Fourteen patients were ventilated; of them 35.7% were expired and most of them required MV more than 7 days (28.7% of 35.7%). These patients developed lung complications due to prolonged mechanical ventilation as nosocomial infections. Comparable to our study, 17% of organophosphate poisoned children in El nagger et al., study [22] developed respiratory failure and necessitated endotracheal intubation and mechanical ventilation; 66.6% of them died. Deterioration of respiratory function was related to excessive secretions, aspiration pneumonia and septicemia complicating adult respiratory distress syndrome [4]. Meticulous monitoring during transport and prompt detection of poor gag reflex could reduce the incidence of aspiration pneumonia. Early recognition of respiratory failure, swift endotracheal intubation, and mechanical ventilation are life-saving measures in severe organophosphate poisoning [25]. In our study, not all poisoned cases with GCS ≤ 8 had been ventilated due to 2 causes. The first is the limited intensive care beds and expensive resources in our locality that necessitate judicious use. The second is some studies that suggested safer approach to observe poisoned patients with decreased consciousness, even if they have a GCS ≤ 8 without intubation. In Duncan and Thakore study [26], none of poisoned patients with GCS of 8 or less suffered aspiration or required intubation. Also, in Sauter et al. [27], study, more than two thirds of intoxicated patients with GCS ≤ 8 were not intubated without any severe complications.

Leucocytosis was significantly higher in non survivors agreeing with several previous studies [28, 29]. Kumar et al. [28], in a prospective observational study found that levels of leucocytes during admission can be used as a prognostic marker in patients with OP poisoning and Tang et al. [29], had also confirmed the diagnostic value of leucocytes in OP poisoning. We detected significantly higher rate of hypoxemia and diminution of Pao2 in non survivors. Respiratory failure was reported to occur in 24-66% of OP poisoning [30–32]. Muscarinic effects of salivation, rhinorrhea, bronchorrhea and bronchospasm and predispose to hypoxemia and increased work of breathing. Nicotinic effects result in muscle weakness and paralysis and contributed to hypercapnic respiratory failure [33]. Regarding type of organophosphate compounds incriminated in the poisoning acts, malathion was the most frequent in our study due to its common use as the least toxic and being an ingredient in local therapies regulated by the United States Food and Drug Administration (FDA) to control head lice [34]. Human have greater carboxylesterase activity relative to levels in insects hence, have the ability to degrade malathion more quickly than it is oxidized to the malaoxon form. This accounts for the relatively low toxicity of malathion [35, 36]. Bioactivation of malathion to the active metabolite malaoxon is necessary to execute its toxic effect through oxidative sulfuration. Toxicity of malaoxon is approximately 22 times more than the parent malathion after ingestion and 33 times more toxic by all other routes of exposure [37]. While we detected malathion as the least lethal organophosphate compound (3.7%), we found chlorpyrifos as the most lethal (22. 2%) agreeing with Liu et al. [38], study where the mortality rate by chlorpyrifos was (15%).

Whatever the situation, diagnosis of OP poisoning must not be delayed and must be picked up basically by history taking, characteristic clinical manifestations, garlic smell of pesticides [39], characteristic clinical presentation, atropine test and decreased serum levels of cholinesterase levels if attainable. In our current study, serum cholinesterase activity was not measured due to non-availability in our center owing to financial impact. However, as a principle, treatment of OP poisoning should be started urgently without waiting the results of serum cholinesterase levels. Rescue workers in primary health institutions should be well trained for provision of emergency services and general population should be informed in regular programs about safer use of pesticides. Moreover, policy towards development of effective and less-toxic pesticides should be warranted.

The strength of this study includes being one of the few studies that give attention to risk factors of mortality due to organophosphates poisoning in Egypt. In addition, this study was a prospective one, so it avoided errors of dependence on previous records and unavailability of some needed data. The most important limitation of this study is the small sample size of studied children. Another important limitation is the lack of cholinesterase activity estimation in our localities whereas the financial matter was an obstacle.

## Conclusion

This study concluded that time consumed till presentation to hospital, low GCS, need for MV, leucocytosis and decreased PaO2 and increased cumulative dose of pralidoxime were independent risk factors of mortality.

## Supplementary Information

**Additional file 1:.** Questionnaire for data collection.

## Data Availability

The datasets used and analysed during the current study are available from the corresponding author on reasonable request.

## Abbreviations

Ach: Acetylcholine
AChE: Acetylcholinesterase
GCS: Glasgow coma scale
MV: Mechanical ventilation
OP: Organophosphates poisoning
OPCs: Organophosphate Compounds
PaCO2: Partial pressures of carbon dioxide
PaO2: Partial pressures of oxygen
