# Supplementary material for: Clinical profile and outcome of acute organophosphate poisoning in children of Upper Egypt: a cross-sectional study
Source: BMC Pediatr. 2021 Feb 26;21:98. doi: 10.1186/s12887-021-02563-w (PMC7908781; doi:10.1186/s12887-021-02563-w)
Supplement: Supplementary file 1 — Additional file 1:. Questionnaire for data collection. [file 12887_2021_2563_MOESM1_ESM.docx]

**Questionnaire for data collection**

1. **Name:**

2.  **Sex** M 🗖 F🗖

1. **Age in years** ≤ 6 years 🗖 >6 - <12years 🗖 ≥ 12 years 🗖
2. **Residence** rural 🗖 urban🗖
3. **Form of organophosphate poisoning**

(specify)------------------------------

1. **Mode of poisoning**  accidental🗖 Suicidal🗖
2. **Route of poisoning** Inhalation🗖 Ingestion 🗖
3. **Identifiable sources of poisoning**

a. Agriculture field

b. Shampoos for head lice

c. Surface & room sprays

d. Spray for house insects

1. **Type of used organophosphate:**
2. **Patient status when coming to the hospital**

conscious🗖 unconscious🗖 Drowsy 🗖 Glasgow coma scale🗖

1. **Time of arrival to the hospital**
2. **Severity of poisoning**

Mild(grade 1) 🗖Moderate(grade 2) 🗖 Severe(grade 3) 🗖

1. **Common clinical manifestations at presentation**

**.............., ..................... .......,......... ................... .....................**

1. **Treatment** Start treatment before arrival 🗖Need for Mechanical ventilation 🗖Decontamination (charcoal &gastric lavage) 🗖Use of atropine 🗖 Use of pralidoxime🗖
2. **Dose of atropine**: Initial at emergency 🗖 cumulative dose 🗖
3. **Dose of pralidoxime:** Initial at emergency 🗖 cumulative dose 🗖
4. Hemoglobin, g/dL🗖 Leukocytes, ×103/mL🗖 Platelets ×103/mL🗖 PaO2 mmHg🗖 PaCO2 mmHg🗖
5. **Duration of hospital stay**----------------------------------------------------------
6. **Outcome/ Improvement** 🗖 **death** 🗖
